# Blended Treatment for Alcohol Use Disorder (Blend-A): Explorative Mixed Methods Pilot and Feasibility Study

**DOI:** 10.2196/17761

**Published:** 2022-04-25

**Authors:** Kristine Tarp, Johan Rasmussen, Anna Mejldal, Marie Paldam Folker, Anette Søgaard Nielsen

**Affiliations:** 1 Centre for Telepsychiatry Mental Health Services Region of Southern Denmark Odense Denmark; 2 Research Unit for Telepsychiatry and E-mental Health Department of Clinical Research University of Southern Denmark Odense Denmark; 3 Steno Diabetes Centre Odense Odense Denmark; 4 Unit of Clinical Alcohol Research Department of Clinical Research University of Southern Denmark Odense Denmark; 5 OPEN Odense Patient data Explorative Network Odense Denmark; 6 Department of Psychiatry Mental Health Services Region of Southern Denmark Odense Denmark; 7 BRIDGE, Brain Research – Inter-Disciplinary Guided Excellence Odense Denmark

**Keywords:** alcohol use disorder, blended treatment, usability, patient perceptions, therapist perspectives, mobile phone

## Abstract

**Background:**

In Denmark, approximately 150,000 people have alcohol use disorder (AUD). However, only approximately 10% seek AUD treatment, preferably outside conventional health care settings and opening hours. The AUD treatment area experiences low adherence to treatment, as well as high numbers of no-show and premature dropouts.

**Objective:**

The purpose of the Blend-A (Blended Treatment for Alcohol Use Disorder) feasibility and pilot study was to describe the process of translating and adapting the Dutch treatment protocol into Danish and Danish culture with a high amount of user involvement and to report how patients and therapists perceived the adapted version, when trying it out.

**Methods:**

The settings were 3 Danish public municipal outpatient alcohol clinics. Study participants were patients and therapists from the 3 settings. Data consisted of survey data from the System Usability Scale, individual patient interviews, and therapist group interviews. Statistical analyses were conducted using the Stata software and Excel. Qualitative analysis was conducted using a theoretical thematic analysis.

**Results:**

The usability of the treatment platform was rated above average. The patients chose to use the blended treatment format because it ensured anonymity and had a flexible design. Platform use formed the basis of face-to-face sessions. The use of the self-determined platform resulted in a more thorough process. Patient involvement qualified development of a feasible system. Managerial support for time use was essential. Guidance from an experienced peer was useful.

**Conclusions:**

This study indicates that, during the processes of translating, adapting, and implementing blended, guided, internet-based, and face-to-face AUD treatment, it is relevant to focus on patient involvement, managerial support, and guidance from experienced peers. Owing to the discrete and flexible design of the blended offer, it appears that it may reach patient groups who would not otherwise have sought treatment. Therefore, blended treatment may increase access to treatment and contribute to reaching people affected by excessive alcohol use, who would not otherwise have sought treatment. In addition, it seems that the blended offer may enhance the participants’ perceived satisfaction and the effect of the treatment course. Thus, it appears that Blend-A may be able to contribute to existing treatment offers. Such findings highlight the need to determine the actual effect of the Blend-A offer; therefore, an effectiveness study with a controlled design is warranted.

## Introduction

### Background

The long-term consequences of alcohol consumption include the risk of developing somatic diseases such as liver cirrhosis, cancer, and cardiovascular disease [1]. Furthermore, untreated alcohol use disorder (AUD) is associated with employment problems, economic burden, high rates of domestic violence, and reduced quality of life [2].

In Denmark, there is general agreement as to what constitutes high-quality treatment of AUD. National and international clinical guidelines recommend evidence-based psychological treatments such as cognitive behavioral therapy (CBT) and motivational interviewing (MI) [3]. Overall, treatment for AUD is easily accessible in Denmark and is free of cost to the patients, in addition to the possibility of patients staying anonymous during the treatment course [4].

The gap between the number of individuals with AUD and those receiving treatment is large [5,6], and those who seek treatment do so when their alcohol dependence is advanced [7]. Among people with persistent AUD, approximately one-fourth do not seek treatment even though they could benefit from it [8]. The reasons for the treatment gap and treatment delay are considered to be that treatment seeking is attached to stigma [9,10], but barriers to treatment seeking also include practical issues, lack of knowledge about treatment, and simply not wishing to stop drinking [11]. Many individuals who seek professional help to curb their alcohol problems have been reported to prefer to receive treatment outside conventional treatment settings and opening hours [12].

Internet-based treatment may be one way to minimize barriers to treatment seeking and increase access to evidence-based treatment for mental health and addictive disorders [13-15]. The potential benefits of internet-based psychological treatment modules include ease of access, cost efficiency, and ability to reach a wide range of users [16]. In particular, guided internet-based treatments have attracted interest during the last decade. Guided internet-based treatment involves a certain level of contact with a therapist, typically via asynchronous chat, text messages, or emails and may function as an important and effective treatment strategy [17,18]. Qualitative studies show that feedback and personal support are perceived positively by patients and keep them motivated [19,20]. Thus, although unguided treatment in some cases may also demonstrate good effect [21], guided internet-based treatment is overall the most promising [22].

Access to personal feedback and to a therapist are features associated with larger effects in a meta-regression of internet-based alcohol interventions [23], and a correlation has been found between personal support in internet-based interventions and positive clinical outcomes and compliance with treatment [24,25]. Departing from this observation, adding internet-based modules to face-to-face treatment [26] or combining face-to-face treatment with internet-based therapy into one integrated and blended treatment protocol has also attracted interest [27-30]. In the blended treatment approach, part of the face-to-face treatment is replaced by internet-based components, while the traditional face-to-face relationship between the therapist and patient is retained. The face-to-face element is considered to ensure that patients benefit from a supportive therapeutic relationship, which is likely to increase their motivation to complete treatment [31,32]. Internet-based elements may provide flexibility, allowing patients access to the treatment modules at the time of their choosing. The increased personal responsibility that comes with such flexibility is also reported to give patients a sense of autonomy and empowerment [33]. Furthermore, through the internet-based platform, therapists can provide feedback to patients and help them stay on track with treatment [31,34].

Pilot studies have found that offering personal support and guidance during web-based addiction treatment in a blended fashion is associated with higher completion levels, increased clinical outcomes, and cost-effectiveness [24,28,35]. Thus, blended treatment may provide a delivery format capable of reaching out to people not inclined to show up for treatment as well as keeping clients motivated during the treatment. In Denmark, however, neither therapist-guided internet-based treatment nor blended treatment for AUD is implemented in the daily clinical routine.

### Blended Treatment at Jellinek

The Dutch addiction treatment clinic, Jellinek, is one of the largest treatment institutions in the Netherlands. Since 2011, Jellinek has been offering blended treatment in routine addiction treatment, and currently, 50% of the patients seek AUD treatment (N=800 per year). Jellinek developed a platform for AUD treatment together with a provider of treatment platforms for internet-based CBT. The platform treatment manual combines face-to-face and internet-based therapy into one integrated protocol [27-29]. The content of the blended protocol is similar to the face-to-face AUD treatment offer, based on evidence-based manuals for MI and CBT [3,36]; the elements are quite similar to the CBT modules in the manual used in project Combine [37]. The blended treatment consisted of a fixed set of sessions, with weekly alternating face-to-face sessions and sessions on the web (approximately 50%:50%) with web-based feedback from the therapist. Sessions on the web were delivered through a secure internet-based treatment platform.

The Jellinek blended treatment approach has not yet been investigated in effectiveness trials, but continuing quality monitoring indicates that treatment compliance is improved when combining face-to-face sessions and internet-based modules with therapist feedback. Dropout rates are lower; patient satisfaction is high; and patients are generally more actively involved in their treatment, spending more time on homework assignments compared with regular face-to-face treatment. Concerning expenditures, Jellinek saw no higher costs per patient compared with regular face-to-face treatment. Furthermore, Jellinek clinics experience increased treatment fidelity to the treatment protocol because of the increased structure in treatment planning and integration of internet-based modules. Thus, although not properly evaluated, the Jellinek blended treatment approach seems to be meaningful, feasible, and promising.

### The Blend-A Study

After a study visit to the Netherlands, we decided to implement and evaluate the treatment approach used at the Jellinek clinics because (1) our impression of the blended treatment protocol was good, (2) the content covered recommendations in Denmark, and (3) both the management and staff at the Jellinek clinics described it as possible to implement in an outpatient treatment structure that is rather similar to the Danish treatment structure. As the Dutch treatment protocol was already digitalized and implemented in the Netherlands, it seemed out of proportion to start from scratch and develop a new treatment protocol to be implemented in Denmark.

As Denmark is *naïve* in making use of internet-based tools in the treatment of AUD, Blend-A (Blended Treatment for Alcohol Use Disorder)—the Blend-A study was developed as an opportunity to evaluate the blended, guided, internet-based, and face-to-face treatment of AUD in Danish settings on a large scale. However, as a predecessor to the Blend-A study, a feasibility study was needed to minimize the need for further adjustments of the treatment platform and work schemes in a large-scale study.

The Blend-A feasibility and pilot study was therefore initiated to develop the Danish version of the Jellinek treatment platform and translate the treatment protocol from Dutch into Danish language and culture; thus, the content of the 2 programs was quite similar. Furthermore, it was initiated to carry out preliminary tests, early phase implementation, and evaluation in 3 AUD treatment clinics. As the research literature typically neither includes a description of the practical process of translating and adapting interventions nor how therapists and patients experience the use of such new internet-based tools, we also decided to collect a series of data in the process, thus making it possible to report our experiences.

### Aim

The aim of this study was to describe the (1) process of developing and adapting the platform and content, (2) therapist and patient experiences of the platform and content during development, (3) implementation of the Blend-A pilot platform, and (4) pilot testing of the platform with new patients and subsequently, to examine the (1) usability of the platform, (2) patient perceptions of a blended treatment course involving the platform, and (3) therapist’s perspectives on blended treatment involving the platform.

## Methods

### Design

The design of the Blend-A feasibility and pilot study was explorative and performed as a mixed methods study [38] based on data obtained from observations, structured questionnaires, semistructured individual patient interviews, and unstructured therapist group interviews.

#### Settings

The Blend-A feasibility and pilot study was conducted in collaboration with three public outpatient alcohol clinics in Southern Denmark: Haderslev, Kolding, and Svendborg municipalities. These clinics are comparable with all publicly funded clinics in Denmark. The treatment was in an outpatient setting, and the staff consisted of a multidisciplinary team of nurses, social workers, and psychiatrists. Therapists are trained in MI and CBT, receive supervision on a regular basis, and follow clinical guidelines. During a normal face-to-face treatment course, patients are initially offered detoxification, if needed. During the acute phase of treatment, they are offered MI and pharmacological treatment, if needed. When withdrawal symptoms have been treated, the patients undergo an assessment interview and are offered individual CBT. Normally, therapy sessions take place every other week and last for approximately 1 hour. The patients underwent status sessions every 3 months, and the treatment course was evaluated. A standard treatment course is planned to last for approximately 3 months but is typically prolonged, if needed [39].

#### Participants

From the 3 clinics, 7 therapists participated in the Blend-A feasibility and pilot study, constantly with 2 from each clinic. In the development and adaptation phase of the Blend-A pilot platform, 3 patients were invited to participate with the therapists. The 3 patients were in the midst of their treatment course, enabling them to contribute to their experiences of what to expect from a treatment protocol. When the Blend-A pilot platform was developed, a series of new, consecutive patients (a total of 20-30 patients were aimed for) from the 3 clinics were invited to participate in the Blend-A pilot study to try the platform during their individual therapy course; 22 of the invited patients agreed to help in the testing and answer the System Usability Scale (SUS) [40]. At the end of the study, 18% (4/22) of these patients were invited and agreed to participate in an individual qualitative interview about their experience of using the pilot platform. At the end of the feasibility and pilot study, the 7 therapists involved in the development, adjustment, and testing were interviewed in group settings about their experiences.

### Platform Development

The development phase was inspired by participatory design [41] and was conducted in an agile process involving therapists and patients in several development and test iterations. This process lasted for 5 months.

#### The Blend-A Pilot Platform and Content

An overview and content description of the platform modules are provided in Table 1. The modules and submodules were organized in a fixed structure. The platform was set up to allow therapists to gradually add sessions on the web to the patient’s individual platform. The sessions on the web contained text and videos with information as well as assignments. According to the treatment protocol, patients received feedback on the web from their therapists on assignments. The platform allows the sharing of information and homework assignments with their significant others. It was estimated that future patients would use the treatment protocol for 3 months and relapse prevention for 6 months. On completion of treatment, future patients will have access to the web-based treatment platform to reread information and look up exercises.

**Table 1:** Blend-A (Blended Treatment for Alcohol Use Disorder) pilot platform modules.

Module number and title and submodule title			Submodule content description
**1. Welcome**			
	Welcome to Blend-A	Explanation of the Blend-A treatment protocol Explanation of the Blend-A research project	
	Onward to a new start	Introduction to being onward to a new start and experience cravings	
	Support from your social network	Introduction to needs for support from social network Task where it can be mapped	
	Test your knowledge on alcohol	Test of knowledge on alcohol	
	Questions and contact	Contact information	
**2. Alcohol treatment**			
	Preparation to change	Explanation of disadvantages when using alcohol and advantages of change Task with the purpose of highlighting the disadvantages of using alcohol and the advantages of quitting drinking Explanation of alcohol registration	
	Goals and techniques for self-control	Explanation of a change plan for alcohol use (goal setting) Explanation of the SMART^a^ criteria Tips for making a change plan Explanation of techniques for self-control concerning alcohol use Task where techniques can be described	
	List of alcohol use risk situations	Explanation of risk situations for alcohol use and instruction to questionnaire Questionnaire where overview >80 risk situations for temptation and self-confidence can be filled out On the basis of the questionnaire, top 5 risk situations are filled out	
	Function analysis and emergency plan	Explanation of function analysis for alcohol use Task: fill out the function analysis Explanation of how an emergency plan can be helpful to prevent relapse or limit the harm Task: description of emergency plans	
	Tackling craving	Description of craving Task: which situations trigger craving, how is craving experienced, and who can craving be explained to Explanation of tasks for 4 different ways to tackle cravings: Diverting yourself by doing something else Surf with your emotions Think differently Talk to your family and friends about it	
	Restructuring	Restructuring of thoughts	
	Turning alcohol offers down	Explanation of how turning down alcohol offers is a skill that can be learnt through role-play Task: description of 3 risk situations and examples of saying no Task: description of a situation where an offer needs to be turned down Task: Role-play—rehearsing turning alcohol offers down Access to diary “evaluation of turning alcohol offers down”	
	Evaluation	Evaluation of turning alcohol offers down	
	Midterm evaluation	Deciding optional skills	
3. Optional skills			Social skills—small talk Social skills—tackling criticism Social skills—giving criticism Tackling feeling sad and depressed Tackling stress Solving problems effectively Tackling relapse
**4. Relapse prevention (maintenance)**			
	Month 1	Alcohol status Quality of life	
	Month 2	Alcohol status Quality of life—your assessment	
	Month 3	Alcohol status Quality of life—your assessment Support	
	Month 4	Alcohol status Quality of life—your assessment Support—your assessment	
	Month 5	Alcohol status Quality of life—your assessment Support—your assessment Motivation	
	Month 6	Alcohol status Quality of life—your assessment Support—your assessment Motivation—your assessment Evaluation of the maintenance phase	

^a^SMART: Specific, Measurable, Achievable, Realistic, and Timely.

#### Data and Data Collection

For patients who agreed to pilot-test the Blend-A pilot platform, data on platform use were retrieved from the platform provider. To assess how the usability developed over time during the pilot study, questionnaire data were retrieved at baseline and 5 follow-ups, with 2 weeks between each measurement. Questionnaire data were collected using a validated Danish version [42] of the questionnaire SUS [40]. The SUS questionnaire consists of 10 questions about a given system’s usability, availability, and coherence. The questionnaire results are presented in Table 2. The 10 questions were answered on a Likert scale from 1 (strongly disagree) to 5 (strongly agree). These 10 responses were used to calculate an SUS score between 0 and 100. Questionnaire data were collected and managed using REDCap (Research Electronic Data Capture; Vanderbilt University) [43], hosted at the Odense Patient data Explorative Network. Questionnaire links and reminders were sent via REDCap every other week to the participants’ private email addresses at baseline and 5 follow-ups, to be filled out on the web using their computer, tablet, or smartphone.

A total of 2 anthropologists and 1 sociologist observed all workshops and training sessions that were performed with patients and therapists throughout the development phase and the implementation and pilot testing of the Blend-A pilot platform and took comprehensive field notes. An anthropologist also conducted qualitative interviews with patients and therapists during the pilot phase.

**Table 2:** System Usability Scale (SUS; Digital Equipment Corporation, 1986).

Items	SUS scores				
	Strongly disagree				Strongly agree

1. I think that I would like to use this system frequently	1	2	3	4	5
2. I found the system unnecessarily complex	1	2	3	4	5
3. I thought the system was easy to use	1	2	3	4	5
4. I think that I would need the support of a technical person to be able to use this system	1	2	3	4	5
5. I found the various functions in this system were well integrated	1	2	3	4	5
6. I thought there was too much inconsistency in this system	1	2	3	4	5
7. I would imagine that most people would learn to use this system very quickly	1	2	3	4	5
8. I found the system very cumbersome to use	1	2	3	4	5
9. I felt very confident using the system	1	2	3	4	5
10. I needed to learn a lot of things before I could get going with this system	1	2	3	4	5

Qualitative data on patient perceptions were collected from 4 individual, semistructured interviews. When the Blend-A pilot platform was implemented in the 3 clinics for half a year and the questionnaire data were collected, patients were invited to participate in the qualitative interviews. The researchers asked the therapists at 2 clinics to invite patients who were willing to use the Blend-A pilot platform and who had answered at least some of the SUS questionnaires. The interviews consisted of open conversations about patients’ experiences with the Blend-A pilot study, supported by an interview guide. Textbox 1 presents the interview guide. The 4 interviews lasted for 14, 19, 24, and 28 minutes, respectively, and were audio recorded and transcribed.

**Interview guide for semistructured individual patient interviews.**

**Interview questions**

- How long have you received blended internet-based and face-to-face treatment?
- How often have you used the platform?
- Why did you use the platform as often or seldom as you did?
- How was it to use the platform?
  to log on
  to read the text
  to solve the assignments
  to receive feedback
- Would you have liked to interact with your therapist via videoconferencing?
- Why did you choose to receive internet-based treatment?
- How was it to receive internet-based treatment, compared with face-to-face treatment?
- What impact has the blended setup had for your interactions with your therapist?
- What impact had the blended setup had on your treatment course?
- Do you have any ideas for alterations?
- Do you have anything to add or ask about?

Additional qualitative data on therapist perspectives were collected from 4 unstructured group interviews conducted with the 7 therapists from the 3 clinics during the pilot study. The group interviews were conducted by an anthropologist and a sociologist. An interview guide updated before each interview inspired the interviews. Textbox 2 presents the interview guide. The group interviews were conducted as part of the implementation process and lasted for approximately 2 hours each. One group interview was audio recorded and transcribed, and field notes were taken from the 3 others.

**Interview guide for therapist group interviews.**

**Interview questions**

- The Blend-A pilot platform
  Which modules have you used?
  Describe your experience of using the platform.
  Describe the patients’ reactions to using the platform.
  How do you experience starting a patient up in the program?
  Is it your impression that the patients feel well informed?
  How is it to give feedback on the platform?
  How do you structure your working week concerning Blend-A?
  Are there functions in the program, which provide new insights in relation to face-to-face sessions?
  Are there insights you do not get when using the Blend-A pilot platform for treatment?
  Have you experienced situations where videoconferencing would have been beneficial?
- Training
  To you who have replaced a therapist: does peer-to-peer training work?
- Workflow description
  How often do you use it?
  Are there workflows we have not described?
  Are there workflows that counteract each other?
  Have you experienced the need of actions outside the platform; for example, mails or calls?
- Supervision and clinical conference
  In your team, how do you consult about the contact to patients using the Blend-A pilot platform?
  Do you have the need for further sparring concerning using the Blend-A pilot platform in your workday?
- Patient recruitment
  How many patients have you recruited?
  How do you recruit?
  What challenges have you found while recruiting for the Blend-A pilot?
  Which reasons do patients provide for choosing to use the Blend-A pilot platform compared with face-to-face sessions?
  Which reasons do patients provide for choosing not to use the Blend-A pilot platform?
- Defects
  What defects have you experienced?
  What defects have the patients expressed?

#### Analyses

Analysis of the rather little structured data from the SUS questionnaires was conducted using the Stata software and Excel.

A qualitative analysis of interviews with patients and therapists was conducted using a theoretical thematic analysis approach [44]. Thematic analysis is a widely used method for identifying and describing themes within data without being bound to any pre-existing theoretical framework. A theme was defined as a meaningful emergence relative to the research question. In the present pilot study, thematic analysis was used as an essentialist or realist method to describe participants’ experiences in the development, implementation, and usability of a blended treatment course involving the Blend-A pilot platform. The analysis consisted of six phases: (1) reading and rereading studies while noting the initial ideas, (2) coding interesting features systematically in the *Results* section, (3) collating codes into potential themes, (4) checking whether the themes work in relation to the coded extracts and entire data set, (5) performing ongoing analysis to refine the specifics of each theme and to generate names for each theme, and (6) performing final analysis of selected extracts relating the analysis to the research question.

Observational data from field notes were used to describe the development process and assess the engagement and concern expressed by both therapists and patients. Similar to the process of analyzing the qualitative interview data, the notes were read, reread, and combined into overall themes and subthemes. In the reporting of the findings from the notes, information stemming from the qualitative interviews of patients and therapists was added to the observational data when they added information about the development process and how the usability and function of the platform was experienced by the parties.

Qualitative analyses were conducted by JR and KT, originally trained as a sociologist and an anthropologist with approximately 10 years of experience each. ASN, with approximately 30 years of experience in qualitative research, supervised the analyses.

### Ethics Approval

The pilot study was notified to the Danish Data Protection Agency (file number 18/1994). The study was conducted in accordance with ethical standards; however, as it is based on questionnaires and interviews, it is not notifiable to the Regional Committees on Health Research Ethics for Southern Denmark. After receiving oral and written information about the project, participants signed consent forms for participation.

## Results

### The Process of Developing and Adjusting Platform and Content

The Blend-A pilot platform is accessible on the web via any given web browser. As mentioned earlier, the platform consisted of 4 modules with submodules, which started with therapy information, followed by multiple exercises and homework assignments, training in optional skills after patients’ individual needs, and relapse prevention.

The Dutch content was maintained overall, as it described MI and CBT-based modules, consistent with standard face-to-face treatment in Danish alcohol treatment clinics. The translation process in which the Dutch platform content was translated into Danish went through five phases: (1) translation of overall concepts and treatment flow was conducted in a full-day workshop together with the platform provider, 3 patients, 6 therapists, 2 consultants, and 2 researchers, of which 1 was a sociologist and 2 were anthropologists; (2) the platform provider made the first rough translation from Dutch to Danish, translating all text, text on buttons, and image texts and providing Danish subtitles on Dutch videos; (3) therapists, researchers, and consultants revised and edited the translation draft, focusing on linguistic, cultural, and therapy-related translations; therapy-related translation was the least time-consuming part, as the included AUD clinics already offered CBT, whereas the linguistic and cultural translation was more time consuming and involved patients, therapists, consultants, and researchers; (4) the content was implemented in a test version of the platform and tested by 6 therapists and 3 patients during a half-day workshop, in particular, during the workshop, the text fields were thoroughly considered and made easy to understand, and in this process, participating patients’ feedback was of particular importance; at the workshop, both therapists and patients went through all elements and discussed the feasibility and acceptability of both the content and layout, and based on this feedback, the content and platform were revised; and (5) the content was then implemented in the production-ready platform, which was named the Blend-A pilot platform.

### Therapist and Patient Experiences of Platform and Content During Development

Patients and therapists who participated in the development and adjustment phases quickly grasped the platform. Both groups provided useful information on the usability of the platform, particularly in spotting passages or larger text blocks, which might be a barrier for future use. The possibilities for flexibilities on how to combine modules were stressed. Both patients and therapists found that videos and graphics were often preferred to text to reduce cognitive load. The videos in the pilot version of Blend-A were Dutch, with the Dutch material subtitled in Danish, and both therapists and patients found that although it might work in a pilot version, it was not optimal. The participating patients became so engaged that in the development process, they kept trying the platform outside the workshop and returned to their therapist with additional information on what they found useful. The therapists found it helpful for future use that they had had the opportunity to work through the content and platform together with patients during the development and adjustment phases, as it allowed them to grasp what engaged the patients the most.

### Implementation of the Blend-A Pilot Platform

During the process of implementing the pilot version, the therapists were trained on how to provide written feedback, patients were recruited, and the Blend-A platform came into operation. The Blend-A pilot platform came into operation in February 2018, and the pilot test was run for 6 months. The therapists involved in the development and translation process described earlier were also the main therapists offering treatment to the new patients via the Blend-A pilot platform. Therapist training before the pilot test consisted of 2 sessions, with 2 hours of training each in the use of the platform and a fortnight of practice in between. A Dutch psychologist experienced in blended treatment participated in preparing the training and offered support to therapists. The therapists were encouraged to offer a blend of face-to-face sessions and internet-based modules that they, together with the patients, found the most helpful and attractive. Thus, no firm structure describing a fixed number of face-to-face sessions before offering the patients the opportunity to continue the treatment course by means of internet-based modules was prescribed. Rather, therapists were encouraged to discuss this with their patients and decide on the optimal blend.

### Pilot Testing of the Platform With New Patients

We planned to pilot test the Blend-A pilot platform with 20-30 new patients. Patients were recruited by means of regular advertisements in local newspapers, Facebook posts, and face-to-face approaches among eligible patients receiving treatment at the 3 clinics. A total of 41 patients completed the Blend-A pilot platform. There was a difference between how far during the treatment program the patients came in the 3 pilot municipalities. In 1 municipality, approximately half of the patients finished the program, and the rest stopped during module 2. In the 2 other municipalities, only one-tenth finished the program, and the rest stopped during and after module 2, respectively. All 41 patients were invited to participate in the evaluation, and 22 agreed to fill out the questionnaires. During the pilot phase and upon request from the therapists, a workflow description for the blended treatment course was developed and regularly updated to provide therapists with details on how to involve a patient in the blended treatment offer. An example workflow is presented in Table 3.

The quantitative and, in particular, qualitative data analyses led to the following findings regarding usability, patient perceptions, and therapist perspectives on the Blend-A pilot platform.

**Table 3:** Example of workflow description.

Patient	Therapist
Contacts the clinic for treatment of AUD^a^.	Offers the patient detoxification, MI^b^, and assessment.
Decides to change habits and work focused.	Offers the patient a treatment course; informs the patient orally about Blend-A and that it is optional to participate, but it requires that the patient has a computer or a tablet; and hands out written information on Blend-A to the patient.
Reads the information sheet at home and decides on participation in Blend-A.	At the first treatment session (flexibility according to resources): asks the patient about participation in Blend-A.
Declines to participate in Blend-A.	Offers patient regular treatment course.
Agrees to participate in Blend-A and signs consent form.	Photocopies the signed consent form and gives the copy to the patient and scans the original form and uploads it to the secure Blend-A Sharepoint.
Receives from and agrees with therapist	Introduces the patient to Blend-A, adds the patient on the platform (administration module can be used by therapists and administrative workers), sends an invitation to the platform to the patient, informs the patient that emails from the platform provider may end up in spam filter, urges patient to store password in a safe place that the patient can remember, agrees with patient on number of sessions internet-based and face-to-face, and informs the patient that it is a possibility to bring a PC to the face-to-face sessions to be introduced to the platform.
Accepts invitation to the platform.	Assigns patient to therapist, assigns treatment modules to the patient, offers to solve some of the first assignments together with the patient, and decides on homework assignments together with the patient.
Uses the platform.	For the rest of the treatment course: receives an email when the patient has solved an assignment, reserves a time slot every week for written feedback on solved assignments (more time consuming in the beginning), and sends reminders to the patient if the assignments are not solved (brief, motivating approach).
Attends treatment session face-to-face.	Completes treatment session with the patient entailing content from the platform.
—^c^	Besides direct patient-therapist interaction: compiles mutual guideline for written feedback, undergoes professional sparring, and participates in treatment conferences.

^a^AUD: alcohol use disorder.

^b^MI: motivational interviewing.

^c^Patient has no task during this step.

### Usability of the Blend-A Pilot Platform

The 22 patients who agreed to participate in the evaluation consisted of 15 (68%) men and 7 (32%) women. Of the 22 patients, 16 (73%) answered one or more SUS questionnaires. A patient was excluded because of a large amount of missing information in the questionnaire. Of the 22 patients, the remaining 15 patients (94%) consisted of 9 (60%) men and 6 (40%) women. Their mean age of the participants was 47 (SD 12) years; the youngest aged 28 years and the oldest aged 73 years.

At baseline, the mean SUS score for the patients (15/22, 68%) was 71 (range 43-85). At the first follow-up, 2 weeks after initiating treatment on the Blend-A pilot platform, the mean SUS score for the patients (10/22, 45%) was 74 (range 53-93). At the second follow-up, the mean SUS score for the patients (3/22, 14%) was 67 (range 58-73). At the third follow-up, the mean SUS score for the patients (3/22, 14%) was 69 (range 63-75). At the fourth follow-up, the mean SUS score for the patients (3/22, 14%) was 78 (range 73-80). At the fifth follow-up, the mean SUS score for the patients (3/22, 14%) was 78 (range 75-85).

### Patient Perceptions of a Blended Treatment Course Involving the Blend-A Pilot Platform

A total of 4 patients who were still enrolled in the treatment agreed to participate in a qualitative interview concerning their experiences with the Blend-A pilot platform. At the time of the conclusion of the pilot study and for the final qualitative interviews, the interviewed patients had been using the Blend-A pilot platform between 3 and 5 months. The interviewed patients used the platform to varying degrees from daily to weekly, typically more frequently at the beginning of the treatment course.

#### Interaction Between Internet-Based and Face-to-Face Treatment

The patients had learned about the possibility of participating in the blended treatment through newspaper advertisements and chose to use Blend-A, particularly because of the blended setup of the treatment course. The platform functioned as a basis for face-to-face sessions with the therapist. Here, a participant explained as follows:

It has been such a good departure point for a talk [...] where she then interprets some things, probably based on my answers [...] so that is kind of like the foundation of it, I think [...] it has worked on me [...] now we have had something to depart from [...] something concrete.

Participant 3, female

It was important for the patients that they could stay at home, read the texts calmly, and solve assignments on the platform. They found that reading the texts started a cognitive process and that performing the assignments touched them emotionally. Being able to use the platform when they had the time to do so had given them breaks, enabling them to think about their answers. Being able to use the platform when they felt motivated had given them the experience of a higher gain from the treatment course, enabling them to apply the therapy to their rehabilitation process, in addition to feeling in control. A participant explained as follows:

I think it is good, this interaction [...] it is very good, and then it is, eh, then I can work with it alone in my head at home in peace and quiet, and then I can come up here [at the clinic] and get a briefing or get a, well what is it called, a pat on the back, that may be able to support me a little to work through some of the stuff again.

Participant 2, female

As such, patients felt that the interaction between the use of the platform and face-to-face sessions had a positive impact on their treatment course.

#### Flexibility

The patients felt that an optimal blend for them was using the platform twice or more per week, together with face-to-face consultations once or twice a month. A patient stated that this cadence was adequate to secure enough substance during the treatment course.

A patient explained that the flexibility in the blended solution was the main reason they enrolled in the treatment. They elaborated on the difference between going to treatment sessions more often and receiving a blended treatment offer with the internet-based platform and fewer face-to-face sessions:

Here, you can do some things at home so you don’t have to spend time on it, there is also a job that needs to be done [...] there are multiple work schedules that, like, has to fit into each other too [...] it is nicest that you can go to and from it and do it when you have the time, or, and when the desire is there [...] it is not something that you have hanging over your head.

Participant 1, male

Because of their work situation, this patient would not have been able to participate in regular treatment with more frequent face-to-face sessions.

#### Anonymity

In addition, the patients liked the possibility of a more anonymous treatment course. A patient explained that it was transgressive to sit in the waiting room, that they felt so wrong, and that it was unpleasant.

Another participant gave anonymity as one of the reasons for choosing the blended treatment course:

It is probably also because it is something that I can sit at home and do, it is a little bit more anonymous, eh, so that was probably why.

Participant 1, male

Another participant preferred the discretion of not attending the clinic more than necessary, as they had the wish not to be recognized as one attending treatment, which could cause feelings of not being able to manage on their own:

I thought that I was drinking too much, and I couldn’t, I couldn’t really succeed in minimizing it and, eh, then, then I saw this offer and an anonymous offer even, and I don’t have to tell anyone so, eh, and I feel...So I thought this is it, now you have to get started with this [...] I could have enrolled treatment in a group with others, and I didn’t feel much like that [...] I liked the anonymous [...] I want to hide myself a little bit and, eh, I don’t want to be seen around by people.

Participant 2, female

However, 2 of them had experienced a shift toward not having the need to remain anonymous.

#### Usability

The patients also emphasized the importance of presenting treatment material on the platform relevant to the patient groups most likely to use Blend-A. It mattered to them that the material was in their native language and targeted to their particular patient group. The patients found it helpful that blended care involved homework assignments, including reading and specific tasks, and that it was possible to go back and repeat the reading and tasks if they felt that they needed a brush up. None of the patients described having shared the content of the platform with their significant others.

For the patients, the platform was simple with regard to the text, examples, assignments, and feedback from therapists. They were satisfied with the platform setup and the reading friendliness of the texts. They found that the platform was easily accessible to use, for example, during the log-on and submission of solved assignments.

However, they emphasized the importance of a range of factors that (1) the platform is technically stable, so it can be used when the needs and wishes are there; (2) it can be seen at first glance that assignments have already been performed; (3) it is easy to return to the assignments and revise; (4) the content is numerically indexed so that feedback can be tracked on the assignments performed; (5) the structure was set up logically; (6) too many questionnaires should be avoided in the modules; and (7) it should be fast to monitor alcohol use, because it is frequently performed. Ideally, the frequency of monitoring should automatically be based on individual needs.

### Therapist Perspectives on Blended Treatment Involving the Blend-A Pilot Platform

The 7 therapists participated in 4 group interviews conducted during the implementation of the Blend-A pilot platform and at the conclusion of the pilot study.

#### Implementation

The therapists emphasized the following factors to be important for training and for future use of the platform: (1) sufficient time for training, that is, reserving one whole day for a training workshop emphasizing case-based training; (2) availability of the therapeutic material in a printed version beforehand, allowing focus on the technical part of the workshop; (3) getting to know the platform properly; that is, working through the platform, pretending to be patients, to understand the program thoroughly from the patients’ views; and (4) in particular, the therapists liked having an experienced peer from the Netherlands describing their experiences with using the platform and giving advice; for example, on how to give written feedback to patients via the platform as this was new to the Danish therapists.

Particularly in the early phase of the implementation of the platform, therapists emphasized the importance of having a workflow description easily available. It was important for them that the document entailed pictures describing various workflows. Moreover, they found it important to have manageable guidelines for maintaining records of the blended treatment courses.

Therapists found that the blended-care approach could easily be integrated into community-based treatment offers for AUD. Providing feedback on patients’ internet-based homework was not considered a problem. On the contrary, the therapists felt that they had more time to reflect and focus compared with giving feedback in a direct face-to-face conversation. In the early days of the pilot phase, therapists allocated 40 minutes a week per patient for written feedback. Later, when they had gotten used to giving feedback in writing, approximately 20 minutes per patient every week was found to be sufficient. In the beginning, the therapists found themselves ever so often logging on to the platform, looking at the assignments handed in, and providing feedback. However, it was their experience that when they gathered it in a dedicated time slot, they gave the patients time to reflect before the new homework was assigned. One of the therapists elaborated as follows:

I am treating a woman over the platform, she’s like 80 years old or something, and she is very industrious and reflective and not one of those who replies in short, but writes a lot, eh, and she stays at home so she has the time to do the assignments, but she, even though she is thorough, she does it quickly, so it is actually me who deliberately has chosen to stall it regarding giving the feedback and say now I will give feedback for three assignments today, and then she can look at that for a few days and then I wait for another couple of days before I give more feedback and then I stall it regarding uploading more so it is me who [...] delays it [...] I think she should have time to reflect in the meantime and not only when she is in front of the screen.

Therapist 1, female

The therapists emphasized managerial support for time use as essential. In addition, they felt that it was important to spend time on (1) educating a dedicated superuser who can allocate time to be the focal point of sparring and information about the use of the platform for treatment, particularly with technical aspects; (2) sparring with colleagues, for example, on how to conduct good blended AUD treatment and provide good feedback; (3) mutual sparring of technical issues when using the platform; and (4) clinical conferences with supervision of blended treatment courses.

#### Usability

The therapists also made several comments on the practicality of the platform. In particular, they suggested not to assign a series of assignments and submodules to the patient simultaneously, but rather to assign them one at a time. They found that the reward for completing a module was important to the patients, and if completing the modules was too time consuming, some patients lost enthusiasm. For those who had not answered the given assignments, therapists sent brief motivating reminders. One of the therapists explained as follows:

We try on Mondays, I give feedback on Wednesdays, so I try to send out like a reminder, and you call it a little shove, I call it something else for them, also to just say that like, you can right there orientate and say, hey where are you, are you a place where you think you need to be discharged.

Therapist 2, female

Thus, the therapists also recommended dividing modules into smaller versions with fewer assignments, which may potentially induce a feeling of more assignment completions and progress for the patients.

Furthermore, the possibility of flexibly combining modules was important. Thus, the therapists recommended allowing assignments to be skipped or new assignments to be released quickly, thereby avoiding waiting times between relevant assignments. They also found that it was important to provide feedback quickly after each module was completed.

#### Patient Recruitment

Concerning the procedure of recruiting patients and motivating them to enroll in a blended treatment course, the therapists emphasized the importance of giving interested patients an opportunity to contact the clinic via email, and not just by telephone or in person, as was the case in the pilot phase. One of the therapists elaborated on their experience of what the patients used the platform for:

Well, it is to stop their alcohol use or at least decrease it [and if this solution had not been a possibility] then they wouldn’t have had any where to turn to, and this is what they say, it is a good way to do it, it is to be able to be totally anonymous if you are, say, a known person in town.

Therapist 5, female

It was their experience that this might make recruitment easier because the contact might feel more anonymous for the patients.

The therapists also experienced how the assignments on the platform were used for extended reflection and served as a foundation for the treatment sessions at the clinic. Here, one of the therapists explained how this may especially be the case for those who feel challenged by sharing in speech:

When he works, it is very clear that things emerge from his assignment-solving that I could not have guessed from our conversations of something, there are some areas for him that he needs to maybe talk about and work more with, and it became very clear, and then I could bring it into our conversations, which made sense for him, so for him it was actually a big help to have something elaborated that he just, which just is but he couldn’t get out into the open on his own, so it is just such a good experience actually, eh, so I think that those patients who don’t say much in the sessions and who think that it might be interesting to solve assignments, it might be an extra bonus in some way.

Therapist 4, female

As different target groups have different needs and wishes for their treatment courses, the therapists suggested offering patients different blends in the future:

1. Anonymous treatment: all initial contacts were handled by email or telephone, and the therapist could only contact the patient by email or by the feedback and message options in the platform. Information about the patient was registered anonymously, and the only information the therapist had about the patient was the patient’s information in the email or in the program.
2. Platform use combined with face-to-face treatment: the patient was treated via telephone or email and was invited to a face-to-face interview. The patient was registered with personal information; that is, their social security number, in the electronic patient record before using the platform. After a month of using the program, there was a follow-up either by telephone or face-to-face conversation.
3. Platform use combined with face-to-face and pharmacological treatment: this blend is similar to face-to-face treatment combined with platform use but with the addition of a clinical treatment consisting of supportive medication. During this treatment form, the patient is followed up more closely every second or third week.

In addition, they emphasized the importance of having a pamphlet to hand out to the patients, describing the blended offer.

## Discussion

### Principal Findings

The purpose of this pilot study was to describe the process of translating and adapting a Dutch treatment protocol on treatment, consisting of blended face-to-face treatment sessions and internet-based modules, into the Danish language and culture. In addition, we report how patients and therapists perceived the adapted version when trying it out. In particular, we focused on experiences from the process of adaptation and pilot testing, which could inform future projects.

Even though the Netherlands and Denmark are much alike, we do have different languages and cultures, as well as slightly differently organized treatment systems. Thus, translating, culturally adjusting, and implementing effective and necessary treatment offers across borders is not straightforward. In this process, it is important to include both therapists and patients to facilitate the development of a treatment program in which they can identify themselves; thus, they choose to complete the program [45,46].

In this study, the development and translation process was inspired by participatory design [41] and was conducted in an agile process involving therapists and patients in several development and test iterations. Such collaboration with end users may more likely result in adaptations considering stakeholder views [41], ensuring the use of the platform and treatment content. Further involvement of patients in the early development phase as well as the later test phases might have helped us to better qualify assessments, videos, and length of the modules, thereby mitigating the need for further refinement of the platform. This is in line with recent qualitative studies concerning web-based alcohol treatment, which emphasized that incorporating patient feedback into the delivery may enable improvement of a treatment offer [45], and that stakeholder feedback can be used to bolster acceptability, appropriateness, and adoption of alcohol internet-based CBT, thus contributing to implementation success [47]. In this study, both patients and therapists were highly engaged in the process and added valuable, in particular pragmatic, information that led to adjustments of both the content and layout of the platform.

We found managerial endorsement and support crucial for the therapists’ dedication of time to the pilot phase, enabling them to recruit patients and provide feedback to the process. Throughout the development process, the 7 therapists each spent 2 hours per week on an average for the project. During the pilot phase, the number of therapists per site (constantly 2 from each clinic) was sufficient to secure a high level of co-ownership in the development of the Blend-A pilot platform. Overall, the therapists participating in this study were very positive toward and engaged in the development and testing of the Blend-A platform. They found that giving feedback to patients in writing, in contrast to the immediate feedback that they were used to giving in face-to-face sessions, led to time to reflect and consider the feedback better. Our findings thus support previous findings on therapists’ experience in delivering guidance via the internet [48]. Previous studies of therapists’ experiences with internet-based CBT have, however, found that therapists may also find it more difficult to *read* patients over the internet [48-50]. Such considerations give support to the praxis of blended therapy, where the patients and therapists have a number of initial face-to-face sessions and then continue working internet-based if it suits the patients’ needs. This study adds to this suggestion.

### Reasons for Seeking Blended Treatment

The pilot study revealed 2 prominent reasons why patients chose to participate in the blended treatment.

The first reason was that the patients were attracted to the blended treatment setup, as it meant that they had to attend the clinic physically less often and at the same time took responsibility for their own treatment. This is in line with findings from other studies on web-based treatments [20,31,51]. In studies on barriers to treatment seeking, factors such as lack of transport and time to attend a treatment course have been found to be structural obstacles to seeking treatment [11,51].

The second reason for seeking a partly internet-based treatment offer was that the patients were attracted to the more anonymous option in the treatment offer owing to the imbedded discretion. We also found that therapists had experienced that allowing for contacting the clinic anonymously by email made patient recruitment easier. During the study, an additional number of patients who did not participate in the Blend-A pilot study were chosen to receive completely anonymous treatment via the Blend-A pilot platform, without face-to-face sessions, to ensure complete anonymity. One of the leading obstacles to seeking treatment is reported to be stigma; for example, because patients feel ashamed to be associated with the clinic and worry about what others might think [9,11,51,52]. This points to the relevance of offering an anonymous version of internet-based alcohol treatment as it might reach a group of non–treatment seekers not otherwise reached, for example, owing to stigma, as also suggested elsewhere [53].

### Platform Use

We found a difference between how far during the treatment program the patients came in the 3 pilot municipalities. In one municipality, approximately half of the patients finished the program, and the rest stopped during module 2. In the 2 other municipalities, only one-tenth finished the program, and the rest stopped during and after module 2, respectively. According to a study on attrition in internet-based treatment of problem drinkers, the challenge of internet-based alcohol treatment programs is no longer their effectiveness but keeping participants involved until the end of the treatment program. In comparison, they found attrition rates of 55% and 65% [54], which seems to be the norm within internet-based alcohol intervention studies [55-57]. It is important to use a significant amount of graphics and videos in the native tongue to reduce the cognitive load. In addition to having an individualized and flexible platform with small modules, it can potentially induce feelings of more assignment completions and progress.

Among the patients, we found a baseline mean SUS score of 71. According to previous research, a system’s usability can be assumed to be above average if the SUS score is ≥68 or higher [58]. We also found a patient-perceived positive impact of the blended treatment course owing to the interaction between platform use and face-to-face sessions, as the former could serve as a basis for the latter. This is in line with findings from other studies on web-based treatments, where it was found that blended therapy might be able to improve treatment usefulness, as it was possible to use input from the platform to prepare for face-to-face sessions [31,34]. However, in the study on blended depression treatment, it was emphasized that face-to-face sessions are crucial to motivate patients and facilitate the guidance of the web-based content [31]. Furthermore, we found a patient-experienced higher gain in the blended treatment, probably stemming from the ability to match treatment needs and the available time to platform use. This finding is concurrent with studies on web-based treatments, where the convenience of always having access to the platform was seen as an enhancer of self-management by enabling the patients to perform the assignments at their own pace [31,51]. In addition, we found rewarding elements in the possibilities of thinking assignments through and revisiting materials on the platform. This finding adds to the findings from previous studies on web-based treatments, where patients have found it easier to express themselves in writing, compared with face-to-face [48,59] or valued being able to download the material to use it after the treatment [45].

We found therapist-experienced importance of reserving a time slot for providing feedback, ensuring both therapist and patient time to work on assignments and feedback. This finding is in agreement with another study [60], which found that therapist feedback expressed a desire to tailor the nature and amount of support to patients. Finally, we found that it was important for the therapists to draw upon the experience of peers during training. Therefore, experiences from the pilot study will be used to inform and train new therapists in the future Danish national rollout of Blend-A.

### Optimal Blend

Patients and therapists participating in the pilot study chose to use the Blend-A pilot platform to varying degrees. Other studies have discussed what might be an optimal blend [27,31]; for example, a study on blended depression treatment [31] found that patients preferred 50% to 60% of the sessions on the web, whereas therapists preferred 75% of the sessions to be face-to-face. The same study also emphasized tailoring treatment to individual patient needs by adjusting the amount and ratio of the web-based modules to patients’ problems, skills, and characteristics. Supporting this, several studies on patient feedback suggest that it may be beneficial to tailor therapist support according to patient needs [32,48,49,61,62]. This might point to the importance of offering different blends targeting the needs of different patient groups, as this might make treatment accessible to an increased number of patients. Hence, the therapists in the present pilot study suggested offering 3- different blends: anonymous treatment, blended treatment, and blended treatment combined with pharmacology.

### Strengths and Limitations

This study is a small feasibility and pilot study and therefore has a series of limitations. First, it was based on a few therapist and patient reports. Although it may be considered a strength that we used a validated questionnaire to assess usability, it is a severe limitation that only 3 patients answered the questionnaire at follow-ups 2 to 5. Therefore, whether the patients’ assessment of the usability of the platform develops over time should be interpreted with caution. In future upscaling, we will rely as much as possible on register data and on more routes for collecting patient data, that is telephone calls and the use of e-Boks (the Danish system for digital communication with the authorities) [63]. Moreover, some usability models are considered problematic, as theory to speculate about the relationship between measures may be lacking [64]. Instead, Drew et al [65] suggested using SUS scores for comparison between, for example, iterations or conjunction with formative usability testing methods to provide a holistic view of real and perceived user experience.

Second, the researchers and consultants who were responsible for the development, adjustment, and implementation of the Blend-A platform worked together and may have been interpreted as being a team by the therapists and patients. Thus, although we do not believe this to be the case, we cannot rule out that this may have led the therapists to be less critical toward the process of development and implementation. It is, however, a strength that 4 group interviews were conducted with therapists, refining their perspectives during the pilot study.

Third, only 4 patients participated in qualitative interviews regarding their experiences with the translated and adapted versions of the platform. We cannot rule out the possibility that those who participated in the qualitative interviews were more positive toward the use of internet-based modules in a treatment course.

Finally, it may be considered a limitation that transcripts and codes of the qualitative interviews were not stakeholder-checked [66] by letting participants provide feedback [67]. However, it is a strength that 3 independent raters were involved in the coding process, as this may enhance the credibility of the analysis [68,69] and increase reliability and internal validity [70].

### Conclusions

Our study indicates that during the processes of translating, developing, and implementing blended, guided, internet-based, and face-to-face AUD treatment, it is relevant to focus on patient involvement, managerial support, and guidance from experienced peers. Owing to the discrete and flexible design of the blended treatment offer, it appears that patient groups who would not otherwise have sought treatment can be reached. Blended treatment may thus increase access to treatment and contribute to reaching people affected by excessive alcohol use, who would not otherwise have sought treatment. Our initial findings indicate that the blended treatment may enhance participants’ perceived satisfaction and the effect of the treatment course. However, we still need to determine the actual effect of Blend-A; therefore, an effectiveness study is currently being performed to evaluate the effect, compliance, and cost-effectiveness of implementing the blended treatment program [63].

## Abbreviations

AUD: alcohol use disorder
Blend-A: Blended Treatment for Alcohol Use Disorder
CBT: cognitive behavioral therapy
MI: motivational interviewing
REDCap: Research Electronic Data Capture
SUS: System Usability Scale
